# Effects of culture on PAMPS/PDMAAm double-network gel on chondrogenic differentiation of mouse C3H10T1/2 cells: in vitro experimental study

**DOI:** 10.1186/1471-2474-15-320

**Published:** 2014-09-27

**Authors:** Yusuke Inagaki, Nobuto Kitamura, Takayuki Kurokawa, Yasuhito Tanaka, Jian P Gong, Kazunori Yasuda, Harukazu Tohyama

**Affiliations:** Department of Sports Medicine and Joint Surgery, Graduate School of Medicine, Hokkaido University, Kita-15 Nishi-7, Sapporo, 060-8638 Japan; Department of Orthopaedic Surgery, Nara Medical University, 840 Shijo-cho, Kashihara, Nara 634-8521 Japan; Laboratory of Soft and Wet Matter, Department of Advanced Transdisciplinary Sciences, Faculty of Advanced Life Science, Hokkaido University, Kita-13 Nishi-8, Sapporo, 060-0810 Japan; Faculty of Health Sciences, Hokkaido University, Kita-12 Nishi-5, Sapporo, 60-0812 Japan

**Keywords:** Cartilage regeneration, Chondrogenesis, Double-network hydrogel, Mesenchymal stem cell

## Abstract

**Background:**

Recently, several animal studies have found that spontaneous hyaline cartilage regeneration can be induced *in vivo* within a large osteochondral defect by implanting a synthetic double-network (DN) hydrogel, which is composed of poly-(2-acrylamido-2-methylpropanesulfonic acid) (PAMPS) and poly-(N,N’-dimethyl acrylamide) (PDMAAm), at the bottom of the defect. However, the effect of hydrogel on hyaline cartilage regeneration remains unexplained. The purpose of this study was to investigate the chondrogenic differentiation of C3H10T1/2 cells on PAMPS/PDMAAm DN gel.

**Methods:**

C3H10T1/2 cells of 1.0 × 10^5^ were cultured on PAMPS/PDMAAm DN gel in polystyrene tissue culture dishes or directly on polystyrene tissue culture dishes. We compared cultured cells on PAMPS/PDMAAm DN gel with those on polystyrene dishes by morphology using phase-contrast microscopy, mRNA expression of aggrecan, type I collagen, type II collagen, Sox 9 and osteocalcin using real-time RT-PCR, and local expression of type II collagen using immunocytochemistry.

**Results:**

C3H10T1/2 cells cultured on the PAMPS/PDMAAm DN gels formed focal adhesions, aggregated rapidly and developed into large nodules within 7 days, while the cells cultured on the polystyrene surface did not. The mRNA levels of aggrecan, type I collagen, type II collagen, Sox 9 and osteocalcin were significantly greater in cells cultured on the PAMPS/PDMAAm DN gel than in those cultured on polystyrene dishes. In addition, C3H10T1/2 cells cultured on PAMPS/PDMAAm DN gel expressed more type II collagen at the protein level when compared with cells cultured on polystyrene dishes.

**Conclusions:**

The present study showed that PAMPS/PDMAAm DN gel enhanced chondrogenesis of C3H10T1/2 cells, which are functionally similar to mesenchymal stem cells. This suggests that mesenchymal stem cells from the bone marrow contribute to spontaneous hyaline cartilage regeneration in vivo in large osteochondral defects after implantation of PAMPS/PDMAAm DN gels.

**Electronic supplementary material:**

The online version of this article (doi:10.1186/1471-2474-15-320) contains supplementary material, which is available to authorized users.

## Background

Articular cartilage injury is a significant and increasing health care concern. It was previously believed that hyaline cartilage tissue cannot regenerate *in vivo*[1, 2]. Recently, however, we found that spontaneous hyaline cartilage regeneration can be induced *in vivo* within a large osteochondral defect in the rabbit at 4 weeks by implanting a synthetic double-network (DN) hydrogel composed of poly-(2-acrylamido-2-methylpropanesulfonic acid) (PAMPS) and poly-(N,N’-dimethyl acrylamide) (PDMAAm) at the bottom of the defect, leaving a 1.5- to 3.5-mm deep vacant space within the defect [3, 4]. The effects of hydrogel on hyaline cartilage regeneration remain unclear. In our pilot study, the PAMPS/PDMAAm DN gel surface enhanced differentiation of chondrogenic ATDC5 cells into chondrocytes *in vitro*. Moreover, Kwon et al. reported that changing the ratio of AMPS and DMAAm could modulate the proliferation and differentiation of chondrogenic ATDC5 cells [5].

Mesenchymal stem cells (MSC) from adjacent bone marrow represent a candidate cell source. There are numerous studies on MSC for cartilage regeneration, as they can be harvested from various tissues and are able to differentiate into cartilage, bone and muscle. Therefore, we believe that investigating the behavior of MSC on PAMPS/PDMAAm DN gel is crucial for the future clinical applications. The C3H10T1/2 cell line was established in 1973 from C3H mouse embryos [6]. Under normal culture conditions, C3H10T1/2 cells display fibroblastic morphology and are functionally similar to MSC. Specifically, these cells develop into osteoblasts, chondrocytes and adipocytes under specific differentiation-inducing culture conditions [7, 8]. This cell line has therefore been widely used in studies of chondrogenesis. Denker et al. reported the chondrogenesis of C3H10T1/2 plating using high-density micromass treatment with bone morphogenetic protein-2 (BMP-2) [9]. The purpose of this study was to investigate the differentiation of C3H10T1/2 cells on PAMPS/PDMAAm DN gel without BMP-2. Our hypothesis was that PAMPS/PDMAAm DN gel is able to enhance chondrogenesis of C3H10T1/2 without BMP-2.

## Methods

### Gel preparation

PAMPS/PDMAAm DN gel was synthesized using the previously reported two-step sequential polymerization method [10]. Briefly, PAMPS hydrogel was obtained by radical polymerization using N,N^’^-methylenebisacrylamide (MBAA) as a cross-linker and 2-oxoglutaric acid was used as an initiator. PAMPS monomer concentration was 1 mol/l, cross-linker concentration was 4 mol% and initiator concentration was 0.1 mol%. Aqueous solution containing monomer, cross-linker and initiator was bubbled with nitrogen for 30 minutes, and was then injected into a cell consisting of a pair of glass plates separated by silicone rubber. The cell was irradiated with a UV lamp (wavelength, 365 nm) for about 6 hours. DN gel was then synthesized by the sequential network formation technique (two-step method). The PAMPS hydrogel (1st network) was immersed in an aqueous solution of 2 mol/L DMAAm, containing 0.1 mol% MBAA and 0.1 mol% 2-oxoglutaric acid for one day until reaching equilibrium. The 2nd network (PDMAAm) was subsequently polymerized in the presence of PAMPS hydrogel by UV irradiation for 6 hours between two plates of glass. After polymerization, the PAMPS-PDMAAm DN gel was immersed in pure water for 1 week and the water was changed twice daily to remove any un-reacted materials. Before cell culture, the gel was punched out at a diameter of 15.4 mm, sterilized by autoclaving (120°C, 20 min), placed in a 24-well polystyrene tissue culture dish, and pre-incubated with maintenance medium overnight.

### Cell culture

The C3H10T1/2 cell line was obtained from the RIKEN cell bank (Tsukuba, Japan). Cells were maintained in Dulbecco’s modified Eagle’s medium (GIBCO, Tokyo, Japan), supplemented with 10% fetal bovine serum, in polystyrene dishes at 37°C under 5% CO_2_. Differentiation medium was prepared by supplementing the maintenance medium with 0.1 μM dexamethasone (Sigma-Aldrich, St. Louis, MO), 0.17 mM ascorbic acid (Sigma-Aldrich), 10 μg/ml human transferrin (Roche Molecular Biochemicals, Mannheim, Germany), 3.0 × 10^-8^ M sodium selenite (Sigma-Aldrich). Maintenance medium or differentiation medium was changed twice a week without damaging the gels.

### Study design

The study was conducted to compare chondrogenic differentiation of C3H10T1/2 cells cultured on PAMPS-PDMAAm DN gels with that on polystyrene dishes. C3H10T1/2 cells (1.0 × 10^5^) were seeded in each well of a 24-well polystyrene tissue culture dish, followed by culture in medium. Cultured cells were observed by phase-contrast microscopy after 7 days of culture (n = 6). We performed real-time polymerase chain reaction (PCR) analyses for gene expression of the markers of chondrocyte differentiation and osteogenesis in cultured C3H10T1/2 cells at 3, 7 and 14 days of culture (n = 6 for each time period). On day 7, we examined the cells for newly formed matrix using immunocytochemistry for type II collagen (n = 4).

All data are given as means and standard deviation. Statistical analyses were performed using analysis of variance (ANOVA) with Fisher’s protected least significance difference test for post-hoc multiple comparisons. A commercially available software program (StatPlus 5.8; AnalystSoft Inc., Vancouver, Canada) was used for statistical calculations. The significance level was set at p = 0.05.

### Real Time RT-PCR analysis

Total RNA was isolated from C3H10T1/2 cells after 3, 7 and 14 days of culture using the RNeasy Mini Kit (Qiagen, Valencia, CA). Quality of total RNA was assured based on A260/280 absorbance ratio determined using a spectrophotometer (NanoDrop Products, Wilmington, DE). Reverse transcription reactions were performed from 0.2 μg of total RNA using PrimeScript RT reagent kit (TakaraBio, Ohtsu, Japan). Real-time PCR for GAPDH, SRY (sex determining region Y)-box 9 (Sox 9), collagen I, collagen II, aggrecan and osteocalcin was conducted using the SYBR green system. Primer sequences are given in Table 1. Real-time PCR was performed using the Thermal Cycler Dice Real Time System (Takara). Samples were held at 95°C for 10 min, followed by 40 amplification cycles consisting of a denaturation step at 95°C for 15 s, and an annulation-extension step at 60°C for 1 min. Gene expression levels were normalized against those of GAPDH.

**Table 1:** **List of primers used in the real-time PCR analysis of gene expression in C3H10T1/2 cells**

Primer ID	Primers (5′-3′)	Amplicon size (bp)	Accession No.
Aggrecan–F	AGTGGATCGGTCTGAATGACAGG	105	NM007424
Aggrecan–R	TTG GCA GCG TTC ATG TCG TAA		
Collagen type I–F	ATGCCGCGACCTCAAGATG	153	NM007742
Collagen type I–R	TGAGGCACAGACGGCTGAGTA		
Collagen type II–F	AGGGCAACAGCAGGTTCACATAC	171	NM031163
Collagen type II–R	TGTCCACACCAAATTCCTGTTCA		
SOX9-F	CAGTACCCGCATCTGCAC	81	NM011448
SOX9-R	TCTCTTCTCGCTCTCGTT		
Osteocalcin-F	CTCTGTCTCTCTGACCTCACAG	133	NM031368
Osteocalcin -R	GGAGCTGCTGTGACATCCATAC		
GAPDH-F	TGTGTCCGTCGTGGATCTGA	150	NM001001303
GAPDH-R	TTGCTGTTGAAGTCGCAGGAG		

### Immunocytochemistry

Immunocytochemistry was also carried out after 1 week of culture. Cells were fixed with 4% paraformaldehyde in phosphate-buffered saline without calcium PBS(-), and were permeabilized with 0.1% Triton X-100 in PBS, followed by pretreatment to block nonspecific reactions with 5% nonimmune goat serum in PBS(-). For type II collagen staining, the primary immunoreaction was carried out with anti-type II collagen antibody (Abcam, Cambridge, UK). Secondary immunoreactions were carried out with Alexa 488-conjugated goat anti mouse IgG (Invitrogen, Carlsbad, CA) in 1% non-immune goat serum in PBS, followed by rinsing with PBS(-). For cell nucleus staining, cells were incubated with 1 μg/ml Hoechst 33258 (Dojindo, Tokyo, Japan) for 1 min. Fluorescence images were observed and recorded with fluorescence microscope (Biorevo BZ-9000; Keyence, Osaka, Japan).

## Results

### Morphological evaluation and immunohistocytochemistry

**Figure 1:**
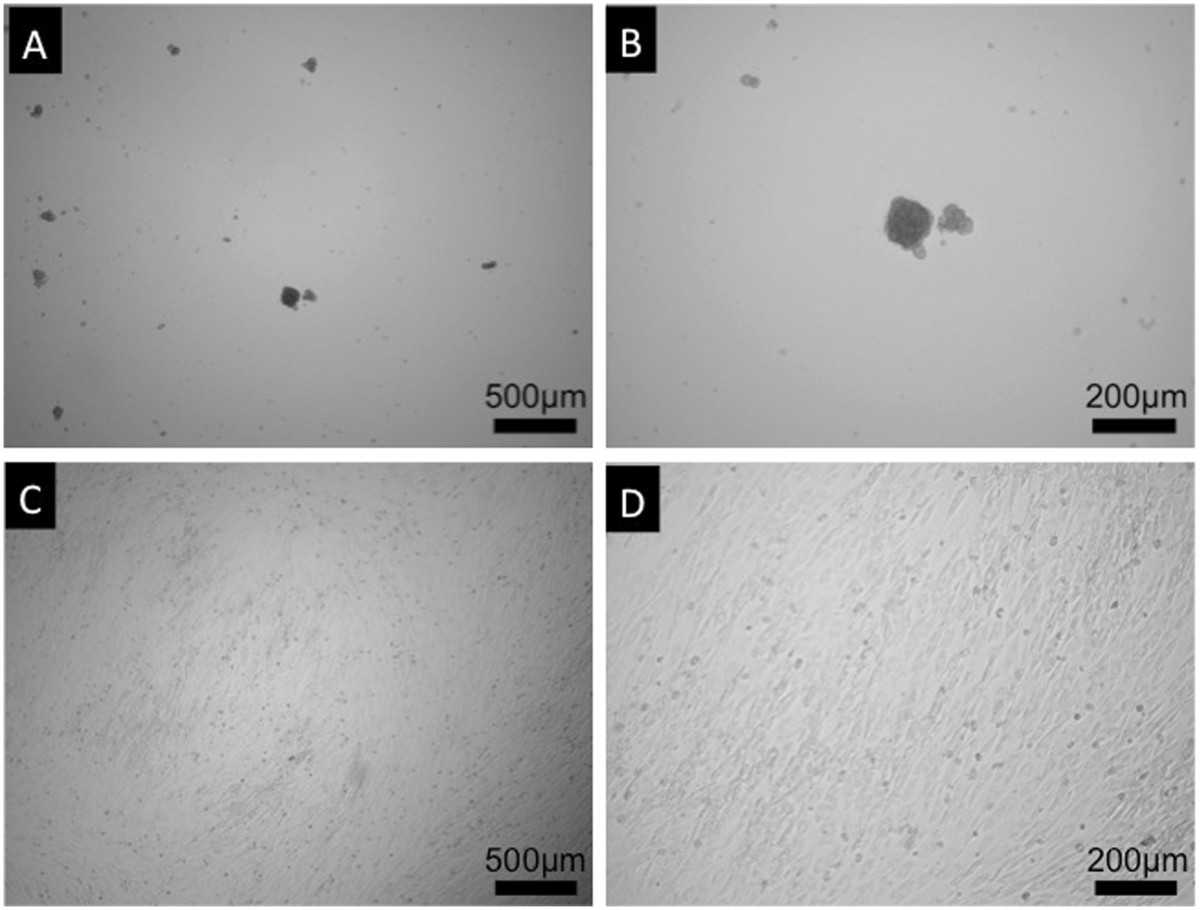
**Phase-contrast microscopy of C3H10T1/2 cells after 7 days of culture.** Cells on PAMPS/PDMAAm DN gels formed nodules (**A**. ×40, **B**. ×100), while cells on polystyrene dishes attached to the substrata (**C**. ×40, **D**. ×100).

**Figure 2:**
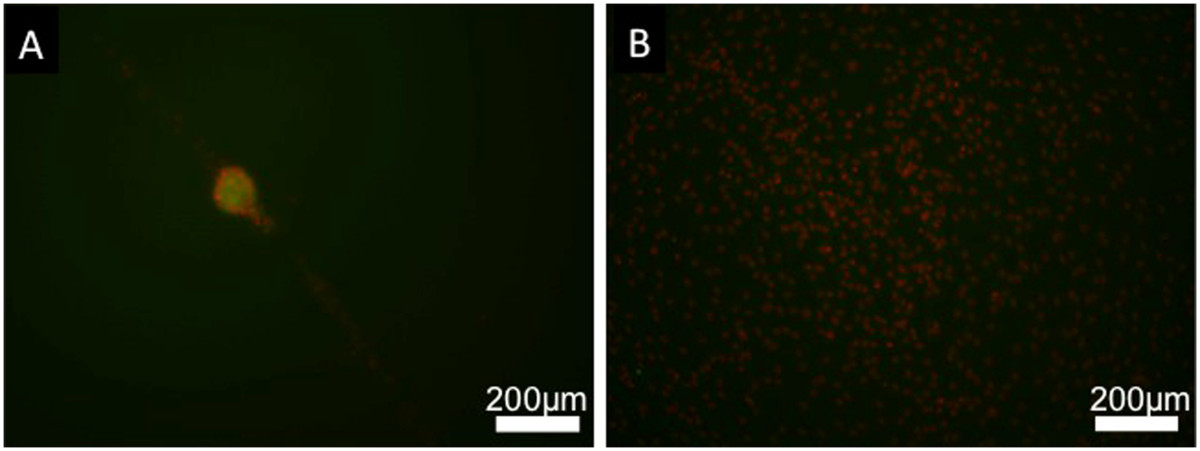
**Expression of type II collagen in C3H10T1/2 cells after 7 days of culture.** Cells were stained with anti-type II collagen antibody (green) and Hoechst 33258 (red). Immunocytochemistry showed obvious expression of type II collagen by cells cultured on PAMPS/PDMAAm DN gels **(A)**, while only weak expression of type II collagen was seen in cells cultured on polystyrene dishes (negative control) **(B)**.

C3H10T1/2 cells, when seeded on PAMPS/PDMAAm DN gels, did not attach to the surface and aggregated with one another to form nodules, which were easily observed macroscopically. Larger nodules did not attach to the surface and floated on the medium (Figure 1-A and B). On the other hand, when cultured on the polystyrene dishes as controls, cells immediately attached to the substrata and continued to proliferate until confluence (Figure 1-C and D). Immunocytochemistry showed obvious expression of type II collagen by cells cultured on the PAMPS/PDMAAm DN gels, and the cells showed weak expression of type II collagen in the peripheral regions of the aggregated cell on polystyrene dishes after 7 days of culture (Figure 2).

### Real-time RT-PCR analysis

**Figure 3:**
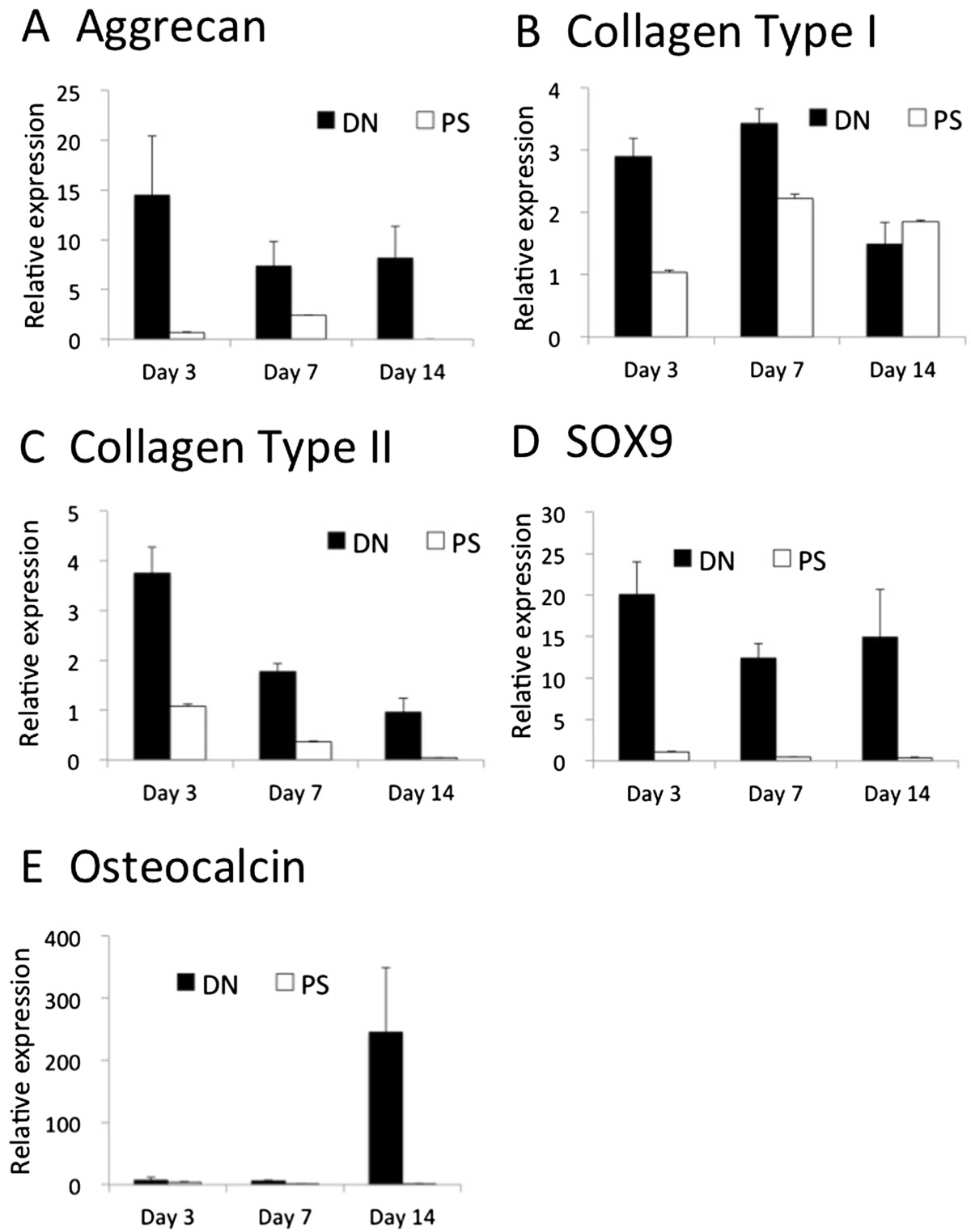
**Gene expression analysis of aggrecan (A), type I collagen (B), type II collagen (C), Sox9 (D) and osteocalcin (E) in C3H10T1/2 cells.** Expression of each gene was measured by quantitative real-time PCR and was normalized against GAPDH expression levels. Values are means ± SEM obtained in five experiments.

ANOVA showed that expression of aggrecan, type I, type II collagens, Sox9 and osteocalcin genes was significantly greater in the cells cultured on the PAMPS/PDMAAm DN gels than on polystyrene dishes (aggrecan: p = 0.00046; type I collagen: p = 0.00002; type II collagen: p = 0.00000; Sox 9: p = 0.00000; and osteocalcin: p = 0.0215). In addition, significant interaction effects between time period and culture condition, i.e., PAMPS/PDMAAm DN gels or polystyrene dishes, were observed on the expression of type I and type II collagen, and osteocalcin genes (type I collagen: p = 0.00006; type II collagen: p = 0.00291; and osteocalcin: p = 0.0175). Aggrecan mRNA levels were significantly greater in cells on PAMPS/PDMAAm DN gels than on polystyrene dishes on days 3 and 14 (Figure 3-A). The expression of type I and type II collagen genes was significantly greater in cells cultured on PAMPS/PDMAAm DN gels than on polystyrene dishes on days 3 and 7 (Figure 3-B and C). The mRNA levels of Sox 9 were significantly higher in cells cultured on PAMPS/PDMAAm DN gels than on the polystyrene dishes at each time period (Figure 3-D), while over-expression of osteocalcin genes was observed in cells cultured on the PAMPS/PDMAAm DN gel on day 14 (Figure 3-E).

## Discussion

We previously reported that PAMPS/PDMAAm DN gel, which we used in the present study, induces hyaline cartilage regeneration within large osteochondral defects after implantation at the bottom of defects *in vivo*[3, 4]. The present *in vitro* study was conducted to examine whether mesenchymal stem cells (MSCs) are able to regenerate hyaline cartilage on PAMPS/PDMAAm DN gels. If MSCs are able to regenerate hyaline cartilage on PAMPS/PDMAAm DN gel, MSCs from adjacent bone marrow would be a candidate cell source in the process of hyaline cartilage regeneration via implantation of PAMPS/PDMAAm DN gel at the bottom of such defects.

The present study showed that C3H10T1/2 cells cultured on PAMPS/PDMAAm DN gels formed focal adhesions, aggregated rapidly and developed into large nodules within 7 days, while cells cultured on the polystyrene surface did not form any nodules during the same period. Our real-time PCR analyses demonstrated significantly greater mRNA levels of aggrecan, type I collagen, type II collagen and Sox 9 than cells cultured on the polystyrene dishes. We also demonstrated that C3H10T1/2 cells cultured on the PAMPS/PDMAAm DN gels expressed type II collagen at the protein level, as compared with cells cultured on the polystyrene dishes. These findings suggest that the PAMPS/PDMAAm DN gels enhanced chondrogenic differentiation of C3H10T1/2 cells, and support the findings of our previous *in vivo* study, which reported spontaneous hyaline cartilage regeneration *in vivo* for a large osteochondral defect after implantation of a plug made from PAMPS/PDMAAm DN gel at the bottom of the defect. The present study also showed that osteocalcin, an osteogenic differentiation marker, was over-expressed in aggregated cells on PAMPS/PDMAAm DN gels after 14 days of culture. Previous investigators showed that three-dimensional aggregation contributes to osteogenic differentiation of mesenchymal stem cells, as demonstrated by the expression of numerous standard osteoblastic markers, including osteocalcin [11]. Long-term aggregation may induce osteogenic differentiation of C3H10T1/2 cells; therefore, we did not attempt to prolong cultures for more than 14 days.

The aggregation of chondroprogenitor mesenchymal cells into precartilage condensation represents one of the earliest events in chondrogenesis. This process is dependent on signals initiated by cell-cell and cell-matrix interactions and is associated with increased cell adhesion and formation of gap junctions and changes in cytoskeletal architecture [12]. There are several possible mechanisms by which PAMPS/PDMAAm DN gels accelerate chondrogenic differentiation in C3H10T1/2 cells. First, the electrostatic properties of the PAMPS/PDMAAm DN gel may enhance precartilage condensation and cell differentiation. We previously reported that ATDC5 cells cultured on highly negatively charged gels, i.e., PAMPS gels and PAMPS-co-DMAAm copolymer gels, formed nodules at day 14, expressing type II collagen and proteoglycan, while ATDC5 cells cultured on the standard polystyrene dish did not differentiate into chondrocytes. Guo et al. also reported that MSCs, when cultured on polystyrene surfaces modified with poly(ethylene glycol) (PEG), did not attach to the surface and aggregated to form pellets immediately after cell seeding. The cells in the pellets had a round morphology and a significant expression of collagen and proteoglycans and the authors concluded that the electrostatic properties of those biomaterials could affect cell differentiation [13]. Second, the PAMPS/PDMAAm DN gel is not only negatively charged, but also has a sulfonic acid base, which is similarly present in proteoglycans in the cartilage matrix. Recent studies have shown that the chemical characteristics of biomaterials influence cell-matrix interactions and play important roles in cell differentiation [14–16]. The PAMPS/PDMAAm gel may thus work as an effective reservoir of signaling molecules or growth factors, providing an appropriate biochemical environment for chondrogenic differentiation of immature cells. Third, the mechanical properties of PAMPS/PDMAAm DN gel may enhance chondrogenic differentiation of C3H10T1/2 [17, 18]. However, in the present study, we found that C3H10T1/2 cells that had seeded on PAMPS/PDMAAm DN gel did not attach to the surface of PAMPS/PDMAAm DN gel. Therefore, it is unlikely that mechanical properties of DN gel enhances chondrogenic differentiation of C3H10T1/2 *in vitro*, and we were unable to confirm the *in vivo* effects of the mechanical properties of DN gel on chondrogenic differentiation of mesenchymal cells.

The limitations of this study were as follows: first, this *in vitro* study does not replicate the mechanical environment of our previous *in vivo* study, in which compression forces were applied to the tissue during weight loading; and second, this cell culture model does not reflect the actual cell supply of the *in vivo* model, in which bone marrow cells around the defect may be sequentially recruited. To elucidate the mechanisms responsible for spontaneous hyaline cartilage regeneration, imaging studies using cell surface markers, determining the cell origin and supply are necessary [19]. In addition, *in vitro* experiments with the addition of cells derived from bone marrow are considered to more closely mirror the *in vivo* situation.

## Conclusion

The present study showed that PAMPS/PDMAAm DN gel enhances chondrogenesis in C3H10T1/2 cells, which are functionally similar to mesenchymal stem cells. This suggests that mesenchymal stem cells from the bone marrow would contribute to spontaneous hyaline cartilage regeneration *in vivo* in large osteochondral defects after implantation of plugs made from PAMPS/PDMAAm DN gel.

## Authors’ original submitted files for images

Below are the links to the authors’ original submitted files for images. Authors’ original file for figure 1 Authors’ original file for figure 2 Authors’ original file for figure 3 Authors’ original file for figure 4

## Abbreviations

AMPS: 2-acrylamido-2-methyl-1-propanesulfonic acid
ANOVA: Analysis of variance
BMP-2: Bone morphogenetic protein-2
DMAAm: N,N’-dimethyl acrylamide
DN: Double-network
GAPDH: Glyceraldehyde-3-phosphate dehydrogenase
MBAA: N,N’-methylenebisacrylamide
MSC: Mesenchymal stem cells
PAMPS: Poly-(2-Acrylamido-2-methylpropanesulfonic acid)
PBS: Phosphate-buffered saline
PCR: Polymerase chain reaction
PDMAAm: Poly-(N,N’-dimethyl acrylamide)
PS: Polystyrene (dish)
SOX9: SRY (sex determining region Y)-box 9
UV: Ultraviolet.
