# Supplementary figures and images for: Effects of culture on PAMPS/PDMAAm double-network gel on chondrogenic differentiation of mouse C3H10T1/2 cells: in vitro experimental study
Source: BMC Musculoskelet Disord. 2014 Sep 27;15:320. doi: 10.1186/1471-2474-15-320 (PMC4190488; doi:10.1186/1471-2474-15-320)

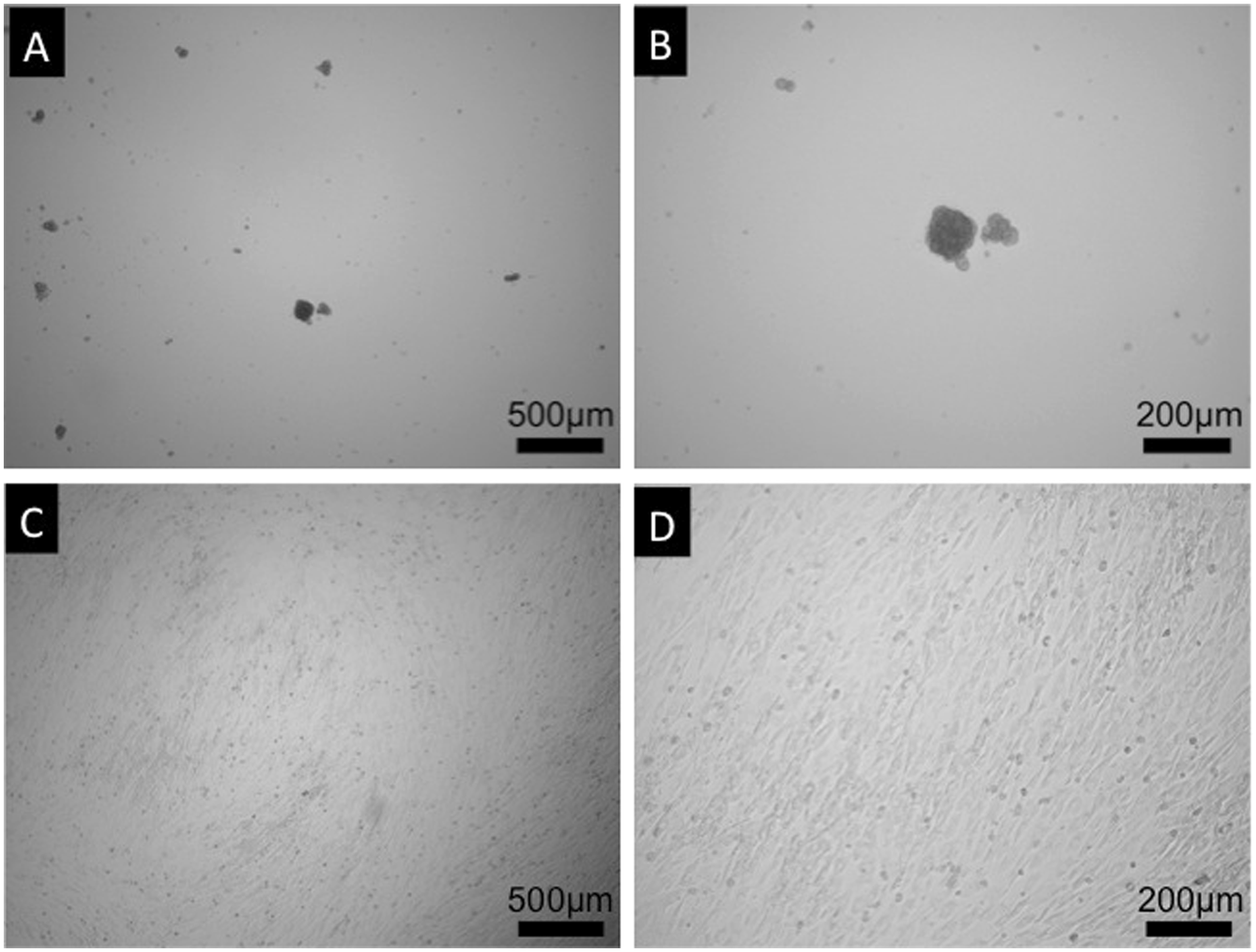

Supplement: Supplementary file 1 — Authors’ original file for figure 1 [file 12891_2014_2257_MOESM1_ESM.tif]

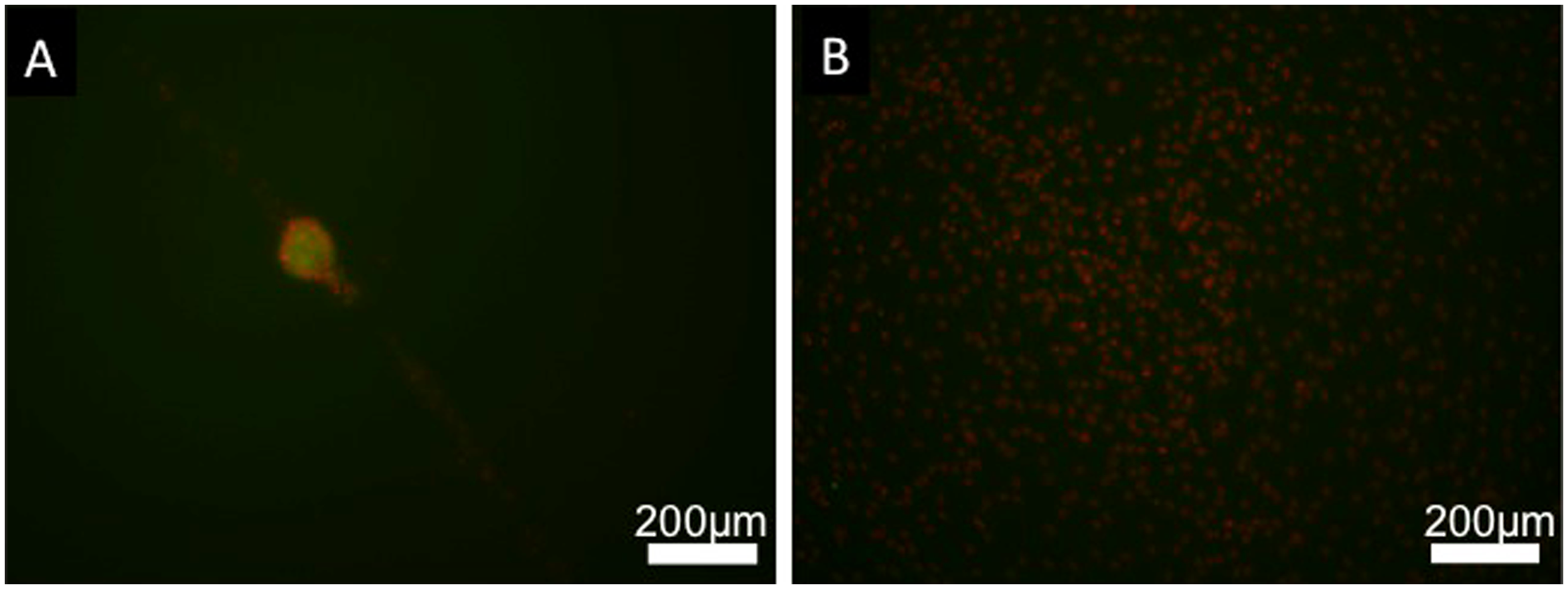

Supplement: Supplementary file 2 — Authors’ original file for figure 2 [file 12891_2014_2257_MOESM2_ESM.tif]

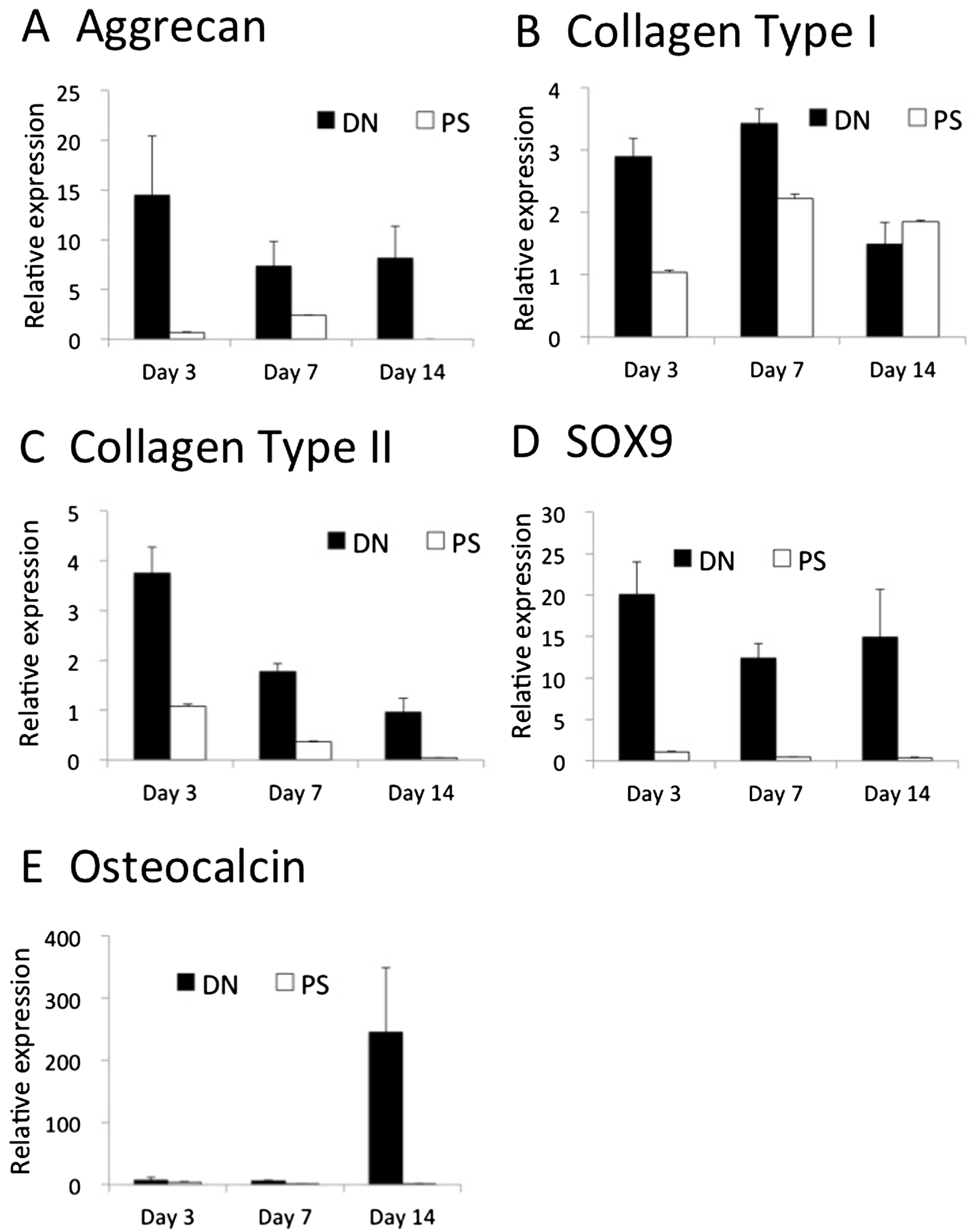

Supplement: Supplementary file 3 — Authors’ original file for figure 3 [file 12891_2014_2257_MOESM3_ESM.tif]
