# Supplementary material for: Effects of culture on PAMPS/PDMAAm double-network gel on chondrogenic differentiation of mouse C3H10T1/2 cells: in vitro experimental study
Source: BMC Musculoskelet Disord. 2014 Sep 27;15:320. doi: 10.1186/1471-2474-15-320 (PMC4190488; doi:10.1186/1471-2474-15-320)
Supplement: Supplementary file 4 — Authors’ original file for figure 4 [file 12891_2014_2257_MOESM4_ESM.docx]

To Reviewer: Maria Prat

1. Indeed a paper on the in vivo properties of the substrate the authors used in this manuscript was already published, although they do not clearly mentioned it (it would be useful to add this reference in the discussion, first paragraph).

Response: According to the reviewer’s advise, we have added the following paragraph to the revised manuscript as follows:

“We previously reported that PAMPS/PDMAAm DN gel, which we used in the present study, induces hyaline cartilage regeneration within large osteochondral defects after implantation at the bottom of defects in vivo [3, 4]. The present in vitro study was conducted to examine whether mesenchymal stem cells (MSCs) are able to regenerate hyaline cartilage on PAMPS/PDMAAm DN gels. If MSCs are able to regenerate hyaline cartilage on PAMPS/PDMAAm DN gel, MSCs from adjacent bone marrow would be a candidate cell source in the process of hyaline cartilage regeneration via implantation of PAMPS/PDMAAm DN gel at the bottom of such defects. (Line 206-)”

1. It is expected that cells aggregate, when they are plated on a cell repellent highly negative substrate. Since a limit of this research, as the authors say, is the fact that they used a fibroblast cell line and not cells derived from bone marrow, and since for the few data presented there are many authors, I would suggest to add also data obtained with bone marrow, which would mirror more closely the in vivo situation.

Response: We agree with this suggestion of the reviewer. The data with addition of cells derived from bone marrow is considered to mirror more closely the in vivo situation. However, it is difficult for the data with addition of cells derived from bone marrow to identify the cell source in the process of hyaline cartilage regeneration within a large osteochondral defect, since the cells derived from bone marrow are heterogenic. We believe that the data of the present study are important to examine whether there is a possibility that mesenchymal stem cells (MSCs) could regenerate hyaline cartilage on the PAMPS/PDMAAm DN gel as the first step for reveling the mechanism of in vivo hyaline cartilage regeneration within a large osteochondral defect by the implantation of he PAMPS/PDMAAm DN gel at the bottom of the defect in vivo. We have added the following comments to the “Discussion” of the revised manuscript.

“The present in vitro study was conducted to examine whether mesenchymal stem cells (MSCs) are able to regenerate hyaline cartilage on PAMPS/PDMAAm DN gels. If MSCs are able to regenerate hyaline cartilage on PAMPS/PDMAAm DN gel, MSCs from adjacent bone marrow would be a candidate cell source in the process of hyaline cartilage regeneration via implantation of PAMPS/PDMAAm DN gel at the bottom of such defects. (Line 208-)”

“To elucidate the mechanisms responsible for spontaneous hyaline cartilage regeneration, imaging studies using cell surface markers, determining the cell origin and supply are necessary [19]. In addition, in vitro experiments with the addition of cells derived from bone marrow are considered to more closely mirror the in vivo situation. (Line 264-)”

1. It would be interesting to prolong the cultures for longer times (e.g. 40-50 days). Do aggregates continue to grow?

Response: For the request from the other reviewer, we evaluated the gene expression of osteocalcin, which is secreted solely by osteoblasts, and found that the cells of the DN-gel group significantly expressed greater level of the osteocalcin gene at 14 days. This finding indicates osteogenesis of C3H10T1/2 cells on the DN-gel at 14 weeks in vitro. Therefore, we did not attempt to prolong the cultures for more than 14 days. We have added the following results and comments in the “Results” and the “Discussion”.

“The mRNA levels of Sox 9 were significantly higher in cells cultured on PAMPS/PDMAAm DN gels than on the polystyrene dishes at each time period (Fig. 3-d), while over-expression of osteocalcin genes was observed in cells cultured on the PAMPS/PDMAAm DN gel on day 14 (Fig. 3-e).(Line 200-)”

“The present study also showed that osteocalcin, an osteogenic differentiation marker, was over-expressed in aggregated cells on PAMPS/PDMAAm DN gels after 14 days of culture. Previous investigators showed that three-dimensional aggregation contributes to osteogenic differentiation of mesenchymal stem cells, as demonstrated by the expression of numerous standard osteoblastic markers, including osteocalcin [11]. Long-term aggregation may induce osteogenic differentiation of C3H10T1/2 cells; therefore, we did not attempt to prolong cultures for more than 14 days. (Line 225-)”

1. In legend of Fig 2 the authors say about proteoglycan staining, but only collagen II staining is shown.

Response: We had examined only collagen II staining in the present study and changed the legend of Fig. 2 as follows:

“Fig. 2. Expression of type II collagen in C3H10T1/2 cells cultured on PAMPS/PDMAAm DN gels (a) and polystyrene dishes (b) after 7 days of culture. Cells were stained with anti-type II collagen antibody (green) and Hoechst 33258 (red).”

1. The legend of Fig. 3 is not clear, I would say not correct, for what it is shown. There is no correspondence with what is shown. The (a) panel is not explained.

Response: According to the reviewer’s advise, we have changed the legend of Fig. 3 as follows:

“Fig. 3. Gene expression analysis of aggrecan (a), type I collagen (b), type II collagen (c), Sox9 (d) and osteocalcin (e) in C3H10T1/2 cells cultured on PAMPS/PDMAAm DN gel (DN) and polystyrene dishes (PS) after 3, 7 and 14 days of culture. Expression of each gene was measured by quantitative real-time PCR and was normalized against GAPDH expression levels. Values are means ± SEM obtained in five experiments. Aggrecan mRNA levels were significantly greater in the cell on DN than on PS on days 3 and 14 (a). Expression of type I and type II collagen was significantly greater in cells cultured on DN than on PS on days 3 and 7 (b and c). mRNA levels of Sox 9 were significantly higher in cells cultured on DN than on PS at each time period (d), while over-expression of osteocalcin genes was observed in cells cultured on DN on day 14 (e).”

1. The figures reported as supplementary data do not add anything, since they are the same presented in the manuscript.

Response: We have shorten the text in the “Results” and add the following brief description of the figures to the revised manuscript.

“Fig. 1. Phase-contrast microscopy of C3H10T1/2 cells cultured on PAMPS/PDMAAm DN gels (DN) (a. ×40, b. ×100) and polystyrene dishes (PS) (c. ×40, d. ×100) after 7 days of culture. C3H10T1/2 cells on PAMPS/PDMAAm DN gels formed nodules (a and b), while cells on polystyrene dishes attached to the substrata (c and d).”

“Fig. 2. Expression of type II collagen in C3H10T1/2 cells cultured on PAMPS/PDMAAm DN gels (a) and polystyrene dishes (b) after 7 days of culture. Cells were stained with anti-type II collagen antibody (green) and Hoechst 33258 (red). Immunocytochemistry showed obvious expression of type II collagen by cells cultured on PAMPS/PDMAAm DN gels (a), while only weak expression of type II collagen was seen in cells cultured on polystyrene dishes (b).”

“Fig. 3. Gene expression analysis of aggrecan (a), type I collagen (b), type II collagen (c), Sox9 (d) and osteocalcin (e) in C3H10T1/2 cells cultured on PAMPS/PDMAAm DN gel (DN) and polystyrene dishes (PS) after 3, 7 and 14 days of culture. Expression of each gene was measured by quantitative real-time PCR and was normalized against GAPDH expression levels. Values are means ± SEM obtained in five experiments. Aggrecan mRNA levels were significantly greater in the cell on DN than on PS on days 3 and 14 (a). Expression of type I and type II collagen was significantly greater in cells cultured on DN than on PS on days 3 and 7 (b and c). mRNA levels of Sox 9 were significantly higher in cells cultured on DN than on PS at each time period (d), while over-expression of osteocalcin genes was observed in cells cultured on DN on day 14 (e).”

1. English is not very good and I recommend a revision for language.

Response: The revised manuscript is proofreaded by a native English speaker. We have attached a Native Check Letter.

To Reviewer: Roland Takács

Reviewer's report:

1. The experiments presented in Fig. 1. and Fig. 2. are not clear in terms of number of independent experiments or repeats. Please clarify.

Response: We had performed 6 independent experiments for observation by phase-contrast microscopy and 4 independent experiments for immunocytochemistry for type II collagen. Therefore, we add the numbers of these experiments in the revised manuscript as follows:

“Cultured cells were observed by phase-contrast microscopy after 7 days of culture (n=6). We performed real-time polymerase chain reaction (PCR) analyses for gene expression of the markers of chondrocyte differentiation and osteogenesis in cultured C3H10T1/2 cells at 3, 7 and 14 days of culture (n=6 for each time period). On day 7, we examined the cells for newly formed matrix using immunocytochemistry for type II collagen (n=4). (Line 139-)”

1. There are numerous spelling mistakes throughout the text, a thorough spell-check would be essential before publication.

Response: We performed a thorough spell-check in the revised manuscript and also received proofreading by a native English speaker.

1. From line 56 the word 'more' or something of similar meaning is missing.

Response: According to the reviewer’s advice, we have changed it as follows:

“In addition, C3H10T1/2 cells cultured on PAMPS/PDMAAm DN gel expressed more type II collagen at the protein level when compared with cells cultured on polystyrene dishes. (Line 60-)”

1. In line 176 'until confluency' would be more appropriate instead of the current phrasing.

Response: According to the reviewer’s advice, we have changed it as follows:

“On the other hand, when cultured on the polystyrene dishes as controls, cells immediately attached to the substrata and continued to proliferate until confluency (Figs 1-c and d). (Line 181-)”

1. There is no verb in the sentence between lines 180-182.

Response: We have changed it as follows:

“Immunocytochemistry showed obvious expression of type II collagen by cells cultured on the PAMPS/PDMAAm DN gels, and the cells showed weak expression of type II collagen in the peripheral regions of the aggregated cell on polystyrene dishes after 7 days of culture (Fig. 2). (Line 183-)”

1. In line 188 'expression of type I gene is written. The word 'collagen' is missing.

Response: We have changed it as follows:

“The expression of type I and type II collagen genes was significantly greater in cells cultured on PAMPS/PDMAAm DN gels than on polystyrene dishes on days 3 and 7 (Figs. 3-b and c). (Line 198-)”

1. Line 208, the correct spelling is 'chondroprogenitor'.

Response: We have changed it as follows:

“The aggregation of chondroprogenitor mesenchymal cells into precartilage condensation represents one of the earliest events in chondrogenesis.(Line 232-)”

1. In line 220 'attached' is written which is grammatically incorrect.

Response: We have changed it as follows:

“Guo et al. also reported that MSCs, when cultured on polystyrene surfaces modified with poly(ethylene glycol) (PEG), did not attach to the surface and aggregated to form pellets immediately after cell seeding. (Line 242-)

1. Please clarify what kind of force does line 233 refer to.

Response: We have changed it as follows:

“However, in the present study, we found that C3H10T1/2 cells that had seeded on PAMPS/PDMAAm DN gel did not attach to the surface of PAMPS/PDMAAm DN gel. PAMPS/PDMAAm DN gel was considered to apply no tensile or compression force to C3H10T1/2 cells. Therefore, it is unlikely that mechanical properties of DN gel enhances chondrogenic differentiation of C3H10T1/2 in vitro, and we were unable to confirm the in vivo effects of the mechanical properties of DN gel on chondrogenic differentiation of mesenchymal cells. (Line 255-)”

1. In Lines 233 and 235 mechanical property should be used in plural form.

Response: We have changed it as follows:

“Third, the mechanical properties of PAMPS/PDMAAm DN gel may enhance chondrogenic differentiation of C3H10T1/2 [17, 18]. However, in the present study, we found that C3H10T1/2 cells that had seeded on PAMPS/PDMAAm DN gel did not attach to the surface of PAMPS/PDMAAm DN gel. Therefore, it is unlikely that mechanical properties of DN gel enhances chondrogenic differentiation of C3H10T1/2 in vitro, and we were unable to confirm the in vivo effects of the mechanical properties of DN gel on chondrogenic differentiation of mesenchymal cells. (Line 253-)”

1. In lines 238 and 239 a verb is missing from the sentence.

Response: We have changed it as follows:

“in which compression forces were applied to the tissue during weight loading; (Line 261-)”

1. In line 242 'determining' should be used instead of 'for'.

Response: We have changed it as follows:

“To elucidate the mechanisms responsible for spontaneous hyaline cartilage regeneration, imaging studies using cell surface markers, determining the cell origin and supply are necessary [19]. (Line 246-)”

1. The word 'to' is missing from the end of line 248.

Response: We have changed it as follows:

“This suggests that mesenchymal stem cells from the bone marrow would contribute to spontaneous hyaline cartilage regeneration in vivo in large osteochondral defects after implantation of plugs made from PAMPS/PDMAAm DN gel. (Line 272-)”

1. In the caption for Fig. 3. there is no part (a), instead (b) is repeated twice.

Response: We have changed it as follows:

“Fig. 3. Gene expression analysis of aggrecan (a), type I collagen (b), type II collagen (c), Sox9 (d) and osteocalcin (e) in C3H10T1/2 cells cultured on PAMPS/PDMAAm DN gel (DN) and polystyrene dishes (PS) after 3, 7 and 14 days of culture.”

1. Classical histological staining procedures that are specific for cartilage extracellular matrix would also support the statements of the authors. Performing some experiments with these stainings (e.g. alcian blue, DMMB, safranin-o-fast-green) would be beneficial to verify the statements made by the authors.

Response: We agree with this suggestion of the reviewer. We tried alcian blue staining for aggregated cells. However, it is difficult for aggregated cells to obtain a satisfactory image with alcian blue stain.

1. In the materials and methods section there are only two temperatures provided for amplification cycles (line 156). Were the annelation temperatures the same as the extension temperature (60 °C) in the case of all genes examined? If no, please clarify.

Response: We set the same temperatue, 60 °C, of the extension to that of annelation for the real time PCR. Therefore, we have changed it as follows:

“Samples were held at 95°C for 10 min, followed by 40 amplification cycles consisting of a denaturation step at 95°C for 15 s, and an annulation-extension step at 60°C for 1 min. (Line 159-)”

To Reviewer: Chun-Yuh Huang

1. Several coating techniques can also prevent cell attachment and promote suspension culture such as agarose coating. An additional control group of suspension culture using other hydrogel should be added for comparison.

Response: We believe that the data of the present study are important to examine whether there is a possibility that mesenchymal stem cells (MSCs) could regenerate hyaline cartilage on the PAMPS/PDMAAm DN gel as the first step for reveling the mechanism of in vivo hyaline cartilage regeneration within a large osteochondral defect by the implantation of he PAMPS/PDMAAm DN gel at the bottom of the defect in vivo. We have added the following comments to the “Discussion” of the revised manuscript.

“The present in vitro study was conducted to examine whether mesenchymal stem cells (MSCs) are able to regenerate hyaline cartilage on PAMPS/PDMAAm DN gels. If MSCs are able to regenerate hyaline cartilage on PAMPS/PDMAAm DN gel, MSCs from adjacent bone marrow would be a candidate cell source in the process of hyaline cartilage regeneration via implantation of PAMPS/PDMAAm DN gel at the bottom of such defects. (Line 208-)”

“To elucidate the mechanisms responsible for spontaneous hyaline cartilage regeneration, imaging studies using cell surface markers, determining the cell origin and supply are necessary [19]. (Line 264-)”

1. Figure 2: A negative control of cell aggregates may be needed as described in last comment.

Response: We have changed it as follows:

“Fig. 2. Expression of type II collagen in C3H10T1/2 cells cultured on PAMPS/PDMAAm DN gels (a) and polystyrene dishes (b) after 7 days of culture. Cells were stained with anti-type II collagen antibody (green) and Hoechst 33258 (red). Immunocytochemistry showed obvious expression of type II collagen by cells cultured on PAMPS/PDMAAm DN gels (a), while only weak expression of type II collagen was seen in cells cultured on polystyrene dishes (negative control)(b).”

1. Figure 3: Explain why gene expression of type II collagen decreased with increasing culture time on PAMPS/PDMAAm DN gel, while gene expression of type I collagen remained unchanged. Did it indicate osteogenesis of C3H10T1/2 cells? Osteogenic gene expression may need to be examined.

Response: We have additionally performed RT-PCR for the gene expression of osteocalcin, an osteogenic differentiation marker, and then found that osteocalcin was over-expressed in aggregated cells on PAMPS/PDMAAm DN gels after 14 days of culture. This suggested that long-term aggregation may induce osteogenic differentiation of C3H10T1/2 cells. Therefore, we have added the following results and comments in the “Results” and the “Discussion”.

“The mRNA levels of Sox 9 were significantly higher in cells cultured on PAMPS/PDMAAm DN gels than on the polystyrene dishes at each time period (Fig. 3-d), while over-expression of osteocalcin genes was observed in cells cultured on the PAMPS/PDMAAm DN gel on day 14 (Fig. 3-e).(Line 200-)”

“The present study also showed that osteocalcin, an osteogenic differentiation marker, was over-expressed in aggregated cells on PAMPS/PDMAAm DN gels after 14 days of culture. Previous investigators showed that three-dimensional aggregation contributes to osteogenic differentiation of mesenchymal stem cells, as demonstrated by the expression of numerous standard osteoblastic markers, including osteocalcin [11]. Long-term aggregation may induce osteogenic differentiation of C3H10T1/2 cells; therefore, we did not attempt to prolong cultures for more than 14 days. (Line 225-)”
